# Retraction to “Protocatechuic acid attenuates cerebral aneurysm formation and progression by inhibiting TNF-alpha/Nrf-2/NF-kB-mediated inflammatory mechanisms in experimental rats”

**DOI:** 10.1515/biol-2022-0808

**Published:** 2023-11-30

**Authors:** Gang Xiao, Mei Zhang, Xing Peng, Guangyuan Jiang

**Affiliations:** Department of Neurosurgery, Chongqing Traditional Chinese Medicine Hospital, No. 6 Panxi 7 Branch Road, Jiangbei District, Chongqing 400021, People’s Republic of China; Department of Dermatology, Daping Hospital, Army Medical University, Chongqing, 400042, China

Retraction of: Gang Xiao, Mei Zhang, Xing Peng, and Guangyuan Jiang. Protocatechuic acid attenuates cerebral aneurysm formation and progression by inhibiting TNF-alpha/Nrf-2/NF-kB-mediated inflammatory mechanisms in experimental rats. Open Life Sciences. Volume 16, Issue 1, DOI: 10.1515/biol-2021-0012.

The authors and the publisher would like to inform that the “Protocatechuic acid attenuates cerebral aneurysm formation and progression by inhibiting TNF-alpha/Nrf-2/NF-kB-mediated inflammatory mechanisms in experimental rats” (https://doi.org/10.1515/biol-2021-0012) has been retracted due to the authors admitting the scientific misconduct in this article.

The authors state as follows:

We realized that the content of the article may not reproducible with the current objectives and focus areas of the research article. This has caused a serious conflicts of interest among authors and research committee of the institution.

- *Figure 2a was wrongly represented and the required finding that “Nrf-2 signaling inhibits intracranial aneurysm formation and progression by modulating vascular smooth muscle cell phenotype and function” was not reproduced in the latest experiments.*
- *The results shown in Figure 2b, c, f and g were not reproduced indicating that the findings were wrongly interpreted with the effect of PCA in rats with H & E staining techniques on the formation and progression of CA. Further, no decrease in the infiltration of the inflammatory cells and reduction in the thickness of the tunica media were observed with the doses of PCA at 50 and 100* *mg/kg, respectively.*
- *Chronic inflammation due to macrophages plays a major role in CA progression, which was inappropriately noticed in Figure 2F that further did not demonstrate staining with anti-CD68 antibodies. This staining shows no positive cells and demonstrates that leukocytes in intracranial aneurysms were not macrophages.*
- *Additionally, Figure 2F did not reproduce numerous leukocytes noticed in intracranial aneurysms and no further changes were noticeable with the treatment of PCA at 50 and 100 mg/kg.*
- *No such claim was reproducible indicating that Nrf-2 expression was significantly decreased in the control group stained with DAB when compared to PCA treated. No difference of expression with the nucleus position in CA (Figure *9*A) was noted in the latest experiments.*
- *Figure 9B depicts that the CA group showed increase in the levels of ROS showing increased fluorescence intensity (3-fold) when compared to the control group, which was substantially (P < 0.05) reduced by the treatment with PCA. No such findings were reproduced to confirm that activation of Nrf-2 causes suppression of intracellular oxidative stress.*

We sincerely apologize for any inconvenience or disruption this may cause to the editorial process. We are aware of the time and effort invested by the reviewers and editors in evaluating our publication, and we genuinely appreciate their considerations. However, we firmly believe that retraction of the article is the most appropriate course of action at this time.

